# Supplementary material for: Experiences and Perspectives of Children and Young People Living with Childhood-Onset Systemic Lupus Erythematosus—An Integrative Review
Source: Children (Basel). 2023 Jun 2;10(6):1006. doi: 10.3390/children10061006 (PMC10297543; doi:10.3390/children10061006)
Supplement: Supplementary file 1 [file children-10-01006-s001.zip › Supplementary material File S1 _ data extraction form.pdf]

Systemic Lupus Erythematosus Integrative Review
**Research Question:**<br>**What are the experiences and perspectives of children and young people living with SLE/lupus/Juvenile onset Systemic Lupus Erythematosus?**
**PEO:**<br>**Population: Children and young people and youth**<br>**Exposure: Living with SLE or lupus**<br>**Outcomes/themes: Experiences/perceptions/perspectives**

Study Description

Study Aim:

Design:

Sample/Participants

Location:

Data /Quotes

Analysis against questions/identified outcomes.

Any other comments:

-
