# Supplementary material for: Experiences and Perspectives of Children and Young People Living with Childhood-Onset Systemic Lupus Erythematosus—An Integrative Review
Source: Children (Basel). 2023 Jun 2;10(6):1006. doi: 10.3390/children10061006 (PMC10297543; doi:10.3390/children10061006)
Supplement: Supplementary file 1 [file children-10-01006-s001.zip › Supplementary Table S1 _Search Architecture.pdf]

Supplementary Table S1. Search architecture in database

Lupus Integrative Review Search Strategy and Results - 3.9.2021

**Database**	**Scopus**
**Key search terms**<br>**Parameters**	(TITLE-ABS-KEY (lupus) AND TITLE-ABS-KEY ((youth OR adolescen* OR "young people" OR "young person" OR teen* OR child* OR kid* OR juvenile OR paediatric OR pediatric) W/8 (perception* OR attitude* OR opinion* OR experience* OR view* OR reflection* OR belief* OR perspective* OR qualitative OR phenomenolog* OR hermeneutic*))) AND PUBYEAR > 1999 AND (LIMIT-TO (LANGUAGE , "English"))
**Total search results**	**313**

**Database**	**CINAHL EBSCO**
**Key search terms**<br>**Parameters**	lupus AND (youth OR adolescen* OR "young people" OR "young person" OR teen* OR child* OR kid* OR juvenile OR paediatric OR pediatric) AND (perception* OR attitude* OR opinion* OR experience* OR view* OR reflection* OR belief* OR perspective* OR qualitative OR phenomenolog* OR hermeneutic*)<br><br>Limited to 2000 – 2021 in English language
**Total search results**	**253**

**Database**	**Medline via PubMed**
**Key search terms**<br>**Parameters**	((lupus) AND (youth OR adolescen* OR "young people" OR "young person" OR teen* OR child* OR kid* OR juvenile OR paediatric OR pediatric)) AND (perception* OR attitude* OR opinion* OR experience* OR view* OR reflection* OR belief* OR perspective* OR qualitative OR phenomenolog* OR hermeneutic*)<br><br>Limited to 01.01.2000 – 01.09.2021
**Total search results**	**947**

**Database**	**PsycInfo via Ovid**
**Key search terms**<br>**Parameters**	# 1 ("systemic lupus erythematosus" or lupus or sle or "juvenile systemic lupus erythematosus").mp. [mp=title, abstract, heading word, table of contents, key concepts, original title, tests & measures, mesh]<br>#2 (youth or adolescen* or "young people" or "young person" or teen* or child* or kid* or juvenile or paediatric or pediatric).mp. [mp=title, abstract, heading word, table of contents, key concepts, original title, tests & measures, mesh]<br>#3 (perception* or attitude* or opinion* or experience* or view* or reflection* or belief* or perspective* or qualitative or phenomenolog* or hermeneutic*).mp. [mp=title, abstract, heading word, table of contents, key concepts, original title, tests & measures, mesh]<br>Limited to 2000 – Current
**Total search results**	Result = 141

**Database**	**Cochrane via Ovid**
**Key search terms**<br>**Parameters**	#1<br>lupus.mp. [mp=ti, ot, ab, tx, kw, ct, hw] (170)<br>#2<br>(youth or adolescen* or "young people" or "young person" or teen* or child* or kid* or juvenile or paediatric or pediatric).mp. [mp=ti, ot, ab, tx, kw, ct, hw] (8,617)<br>#3<br>(perception* or attitude* or opinion* or experience* or view* or reflection* or belief* or perspective* or qualitative or phenomenolog* or hermeneutic*).mp. [mp=ti, ot, ab, tx, kw, ct, hw] (12,507)<br>#1 AND #2 AND #3 (100)<br>Limited to 2000 – current
**Total search results**	**79**

1,715 in total
