# Supplementary material for: Experiences and Perspectives of Children and Young People Living with Childhood-Onset Systemic Lupus Erythematosus—An Integrative Review
Source: Children (Basel). 2023 Jun 2;10(6):1006. doi: 10.3390/children10061006 (PMC10297543; doi:10.3390/children10061006)
Supplement: Supplementary file 1 [file children-10-01006-s001.zip › Supplementary Table S2_MMAT combined results.pdf]

Table S2. Mixed Methods Appraisal Tool (MMAT) Combined Results															
**Author, year (Ref.)**	**Qualitative Studies**					**Quantitative Descriptive Studies**					**Mixed Methods Studies**				
	1.1.	1.2.	1.3.	1.4.	1.5.	4.1.	4.2.	4.3.	4.4.	4.5.	5.1.	5.2.	5.3.	5.4.	5.5.
Beckerman, N., & Sarracco, M. (2012)	Yes	Yes	Yes	Yes	Can't tell										
Brunner, H. I., et al., (2009)						Yes	Yes	Yes	Yes	Yes					
Case, S., et al., (2021).	Yes	Yes	Yes	Yes	Yes										
Ceppas Resende, et al ., (2016)	Yes	Yes	Yes	Yes	Yes										
Davis, A. M., et al., (2018)						Yes	Yes	Yes	Yes	Yes					
Donnelly, C., et al., (2018)						Yes	Yes	Yes	Yes	Yes					
Harry, O., et al.,(2019)											Yes	Yes	No	No	No
Hernández Zapata, L. J., et al., (2018)	Yes	Yes	Yes	Yes	Yes										
Ji, L.,et al., (2012)						Yes	Yes	Yes	Yes	Yes					
Jones, J. T., et al., (2016)						Yes	Yes	Yes	Yes	Yes					
Kohut, S., et al., (2013).						Yes	Yes	Yes	Yes	Yes					
Moorthy, L. N.,et al., (2010)						Yes	Yes	Yes	Yes	Yes					
Moorthy, L. N., et al., (2004)	Yes	Yes	Yes	Yes	Yes										
Moorthy, L. N., et al.,(2017)						Yes	Yes	Yes	Yes	Yes					
Ruperto, N., et al., (2004)						Yes	Yes	Yes	Yes	Yes					
Smith, E. M. D., et al.,(2021)	Yes	Yes	Yes	Yes	Yes										

Tan, Q. E. C., et al., (2021)	Yes	Yes	Yes	Yes	Yes					
Tunnicliffe, D. J., et al., (2016)	Yes	Yes	Yes	Yes	Yes					
Uzuner, S., et al., (2017)						Yes	Yes	Yes	Yes	Yes
